# Kinetics of protein-assisted nucleic acid interconversion monitored by transient time resolved fluorescence in microfluidic droplets

**DOI:** 10.1093/nar/gkab687

**Published:** 2021-08-27

**Authors:** Natalia Grytsyk, Damien Cianfarani, Olivier Crégut, Ludovic Richert, Christian Boudier, Nicolas Humbert, Pascal Didier, Yves Mély, Jérémie Léonard

**Affiliations:** Institut de Physique et Chimie des Matériaux de Strasbourg, Université de Strasbourg & CNRS, 67034 Strasbourg, France; Laboratoire de Bioimagerie et Pathologies, UMR 7021 CNRS, Université de Strasbourg, Faculté de Pharmacie, 67401 Illkirch, France; Institut de Physique et Chimie des Matériaux de Strasbourg, Université de Strasbourg & CNRS, 67034 Strasbourg, France; Institut de Physique et Chimie des Matériaux de Strasbourg, Université de Strasbourg & CNRS, 67034 Strasbourg, France; Laboratoire de Bioimagerie et Pathologies, UMR 7021 CNRS, Université de Strasbourg, Faculté de Pharmacie, 67401 Illkirch, France; Laboratoire de Bioimagerie et Pathologies, UMR 7021 CNRS, Université de Strasbourg, Faculté de Pharmacie, 67401 Illkirch, France; Laboratoire de Bioimagerie et Pathologies, UMR 7021 CNRS, Université de Strasbourg, Faculté de Pharmacie, 67401 Illkirch, France; Laboratoire de Bioimagerie et Pathologies, UMR 7021 CNRS, Université de Strasbourg, Faculté de Pharmacie, 67401 Illkirch, France; Laboratoire de Bioimagerie et Pathologies, UMR 7021 CNRS, Université de Strasbourg, Faculté de Pharmacie, 67401 Illkirch, France; Institut de Physique et Chimie des Matériaux de Strasbourg, Université de Strasbourg & CNRS, 67034 Strasbourg, France

## Abstract

Interconversions between nucleic acid structures play an important role in transcriptional and translational regulation and also in repair and recombination. These interconversions are frequently promoted by nucleic acid chaperone proteins. To monitor their kinetics, Förster resonance energy transfer (FRET) is widely exploited using ensemble fluorescence intensity measurements in pre-steady-state stopped-flow experiments. Such experiments only provide a weighted average of the emission of all species in solution and consume large quantities of materials. Herein, we lift these limitations by combining time-resolved fluorescence (TRF) with droplet microfluidics (DmF). We validate the innovative TRF-DmF approach by investigating the well characterized annealing of the HIV-1 (+)/(–) Primer Binding Sequences (PBS) promoted by a HIV-1 nucleocapsid peptide. Upon rapid mixing of the FRET-labelled (–)PBS with its complementary (+)PBS sequence inside microdroplets, the TRF-DmF set-up enables resolving the time evolution of sub-populations of reacting species and reveals an early intermediate with a ∼50 ps donor fluorescence lifetime never identified so far. TRF-DmF also favorably compares with single molecule experiments, as it offers an accurate control of concentrations with no upper limit, no need to graft one partner on a surface and no photobleaching issues.

## INTRODUCTION

Though the primary structure of nucleic acids (NAs) is rather simple, being just based on four nucleobases, their secondary and tertiary structures could be much more complex. Secondary structures mainly result from base pairing between complementary strands to form duplexes. In single stranded sequences, base pairing can lead to the formation of stems that are frequently associated with loops, bulges, mismatches, and junctions (1–4). Moreover, backbone-backbone interaction together with non-canonical and canonical base pairing can provide tertiary structures such as kissing loops, triple helices, G quadruplexes, i-motifs, cruciforms or pseudoknots. These structures frequently interconvert, being energetically close. This structural polymorphism is an intrinsic property of NAs which results from their rugged energy landscape and allows them to exert multiple functions in transcriptional and translational regulation, recombination, repair or viral infections (1,5,6). Interconversion between different structures depends on physico-chemical factors that include temperature, base composition, salt concentrations, and ligands. It can also be guided by NA chaperone proteins (7) that are multifunctional proteins rich in arginine and intrinsically disordered structural regions. These proteins transiently bind to a wide range of NA sequences and conformations, and destabilize the less stable structures through an entropy transfer mechanism, which in turn promotes the formation of the most stable conformation (8–10). NA chaperones can notably promote the annealing of complementary sequences to form a duplex. (11–15) This activity is especially important when the initial sequences are folded in stable structures such as stem-loops.

Elucidating the mechanisms governing the annealing of complementary NA sequences promoted by NA chaperones is challenging because these reactions are frequently rapid and involve transient complexes that cannot be isolated. Moreover, as the final duplex is much more stable than the initial reactants, the reaction is almost irreversible and needs to be monitored in out-of-equilibrium—also called pre-steady-sate—conditions. Due to their exquisite sensitivity and spatio-temporal resolution, fluorescence-based techniques and in particular techniques based on Förster resonance energy transfer (FRET) appear highly suited to monitor these reactions in real time and resolve their mechanisms. Indeed, FRET efficiency being dependent on the distance and angular orientation between the FRET donor/acceptor pair, FRET experiments are well suited to monitor the conformational transitions of the labelled oligonucleotide during the annealing process. Intensity-based, ensemble FRET experiments have been amply used to investigate annealing kinetics in stopped-flow experiments producing pre-steady-state initial conditions to investigate irreversible reaction kinetics. These measurements are simple and robust, but suffer from the fact that fluorescence intensity is a weighted average of the emission of all species in solution. As a result, the emission and the concentrations of the intermediate complexes cannot be directly visualized, but can only be inferred from the fits of the kinetic traces with a given kinetic model.

Multiple species—i.e. structural heterogeneity—can be resolved in FRET experiments provided one performs single molecule (SM) spectroscopy or time-resolved (TR) fluorescence spectroscopy (see the list of abbreviations in the SI). While SM-FRET experiments are ideally evidencing structural heterogeneity and interconversion kinetics, (16) many individual experiments are required to achieve a statistically relevant description of the relaxation kinetics of sub-populations. Conversely, TR-FRET are ensemble experiments providing statistically relevant distributions of fluorescence decay times—corresponding to distributions of donor-acceptor distances—with relative decay amplitudes revealing the concentration of the different species, but no information about interconversion kinetics (17–21). These techniques are well-suited to investigate equilibrium interconversion rates or distributions, respectively, but require further refinements to investigate irreversible reaction kinetics, starting from pre-steady-state conditions.

To monitor structural heterogeneity in pre-steady-state conditions, *transient* Time-Resolved FRET was successfully achieved by combining time-resolved FRET (TR-FRET) with a conventional stopped-flow apparatus to investigate the structural kinetics of myosin, but this approach consumed high quantities of protein (22). Microfluidics technology is a priori solving the issue by enabling fast mixing times and very low material consumption. While TR-FRET was combined with rapid, continuous-flow microfluidic mixers (23) to investigate e.g. protein folding on the microsecond time scale, (24,25) droplet microfluidics (DmF) appears better suited to investigate (bio)chemical reactions on slower time scales (26–29). Indeed, DmF produces water-in-oil droplets—i.e. microreactors—where several reagents can be mixed on the millisecond time scale, and which can then be propagated or stored in microfluidic devices over seconds to hours, thus enabling the monitoring of irreversible reaction kinetics on such time scales. For these reasons, we combined DmF with TRF detection to implement transient TRF and demonstrated that this approach—hereafter called TRF-DmF—enables monitoring the structural evolution of pre-steady-state biomolecular systems (30). As a proof-of-principle experiment we monitored the fluorescence decay kinetics of Patent Blue V—a fluorescent probe for local viscosity—upon mixing and binding with Human Serum Albumin within ∼100 picolitre droplets propagating over centimeters (i.e. seconds) in a microfluidic channel.

Here, we apply the TRF-DmF experimental approach to monitor—by TR-FRET—the annealing of complementary NA sequences promoted by a NA chaperone protein. As a model system, we used the well characterized annealing of the HIV-1 (+)/(–) Primer Binding Sequences (PBS) promoted by the HIV-1 nucleocapsid NC(11–55) peptide, acting as a NA chaperone. This system mimics a key step of the HIV-1 reverse transcription process (31,32). Using fluorescence intensity (FI) detection, NC(11–55) was previously shown to efficiently promote the (+)/(–)PBS annealing reaction through a two-step reaction involving a kissing loop intermediate (33–35). This intermediate was inferred from the kinetic traces but never directly observed. By using TRF-DmF to monitor the reaction of (–)PBS doubly labelled by fluorescein and the fluorescence quencher dabcyl with its complementary (+)PBS sequence, we could for the first time simultaneously follow the time evolution of the populations of free stem-loop (SL) (–)PBS and the intermediate complexes (IC’s). In particular, we observed an early IC structure associated with a very short donor fluorescence lifetime (∼50 ps), thus providing an unprecedented, direct evidence and structural information for this initial transient complex. The significance of our work is twofold. First, we demonstrate the strength of the TRF-DmF approach for kinetic NA conversion studies as compared to Transient Fluorescence Intensity (Tr-IF) experiments (e.g. conventional stopped-flow) where IC’s may not be observed directly and too-weakly fluorescent species remain unnoticed. Second, our data allow us to propose a refined model for the NC(11–55)-promoted (+)/(–)PBS hybridization reaction, where two successive IC's are identified and structurally characterized.

## MATERIALS AND METHODS

### Mixing experiments with the TRF-DmF set-up

**Scheme 1.**
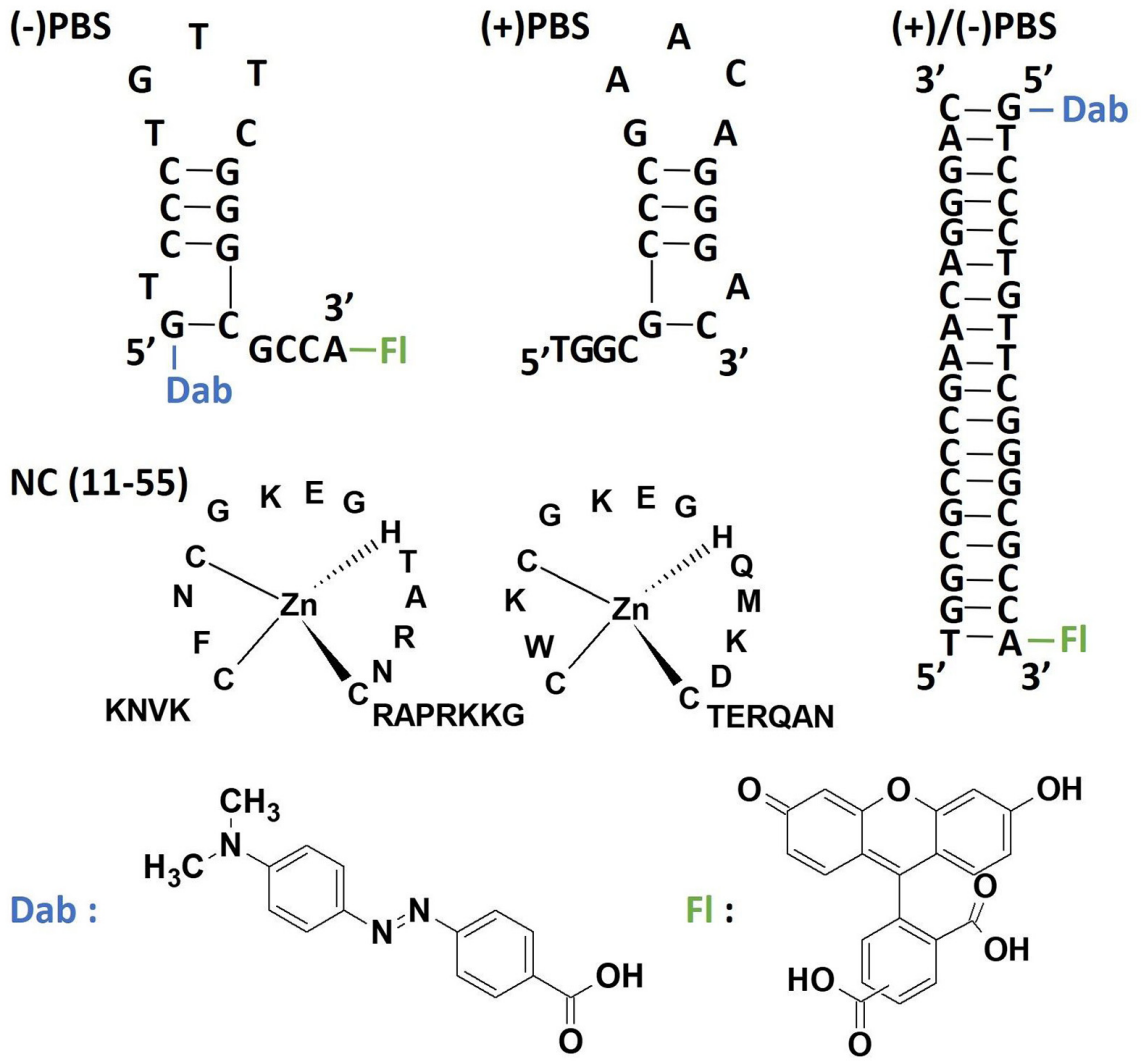
Structures of the PBS sequences, Dab and Fl fluorescent labels, and NC(11–55) peptide.

In order to monitor the (+)/(–)PBS hybridization reaction by transient TR-FRET, we propose to use droplet microfluidics to (i) mix rapidly (in a few ms, see (30)) (–)PBS and its complementary (+)PBS sequence and (ii) follow the structural relaxation of the pre-steady-sate complex as a function of the droplets propagation time along the main channel downstream the mixing region. The (–)/(+)PBS hybridization reaction thus occurs within the droplets serving as micro reactors which propagate along the main microfluidic channel as a function of time. The structural information about the biomolecular complex is encoded by the distribution of fluorescence lifetimes of 5(6)-carboxyfluorescein (Fl or 5(6)-FAM) covalently linked on the 3′ end of the hairpin-shaped (–)PBS, in the presence of 4-(dimethylaminoazo)benzene-4-carboxylic acid (Dab or dabcyl) covalently linked at its 5′ end, acting both as a fluorescence quencher and FRET acceptor. In the doubly-labelled (–)PBS_5′Dab_3′Fl, the Fl emission is quenched by Dab because the distance between the two probes is short enough in the hairpin structure (see Scheme 1). Upon hybridization with the complementary, non-labelled (+)PBS sequence, the distance between donor and quencher increases due to the formation of the extended duplex (ED). Therefore, the donor fluorescence intensity and average lifetime increase. Here, rather than detecting the fluorescence intensity or average lifetime, we aim at characterizing the *distribution* of fluorescence lifetimes and its temporal evolution along the course of the hybridization reaction, by implementing time-resolved fluorescence (TRF) spectroscopy along a microfluidic channel using a microscope equipped with a streak camera.

**Figure 1.**
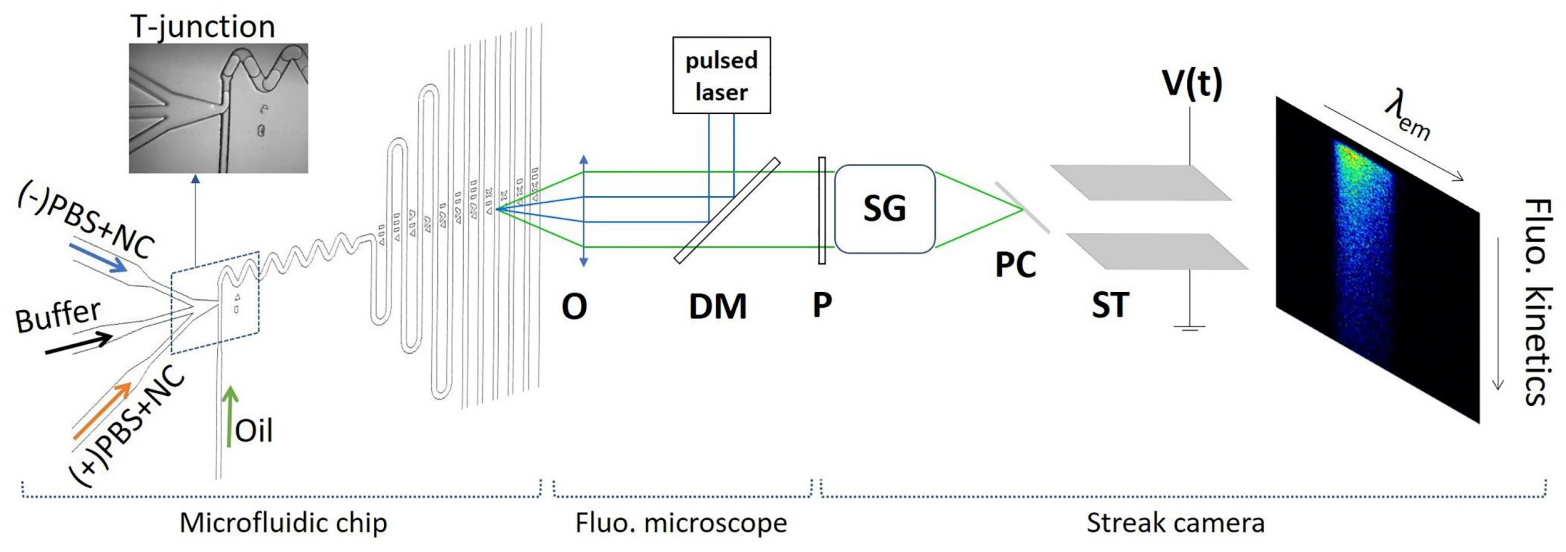
Scheme of the TRF-DmF experimental setup. A scheme of the microfluidic chip is displayed on the left hand side with a microphotograph (in insert) of the T-junction where water-in-oil droplets are formed. An in-house developed fluorescence microscope is used to excite and collect time-resolved fluorescence along the main microfluidic channel (O: microscope objective, DM: dichroic mirror, P: Polarizer). The Fluorescence emission from droplets is imaged through a spectrograph (SG) on the photocathode (PC) of a streak camera (ST: streak tube). The fluorescence signal is resolved spectrally (horizontal axis of the PC) and temporally (vertical axis of the ST).

The actual implementation of this TRF-DmF experimental approach (Figure 1) was already demonstrated and described in detail in ref (30). The microfluidic chip design follows that of Ismagilov and coworkers (26). A T-junction configuration is employed to produce water-in-oil droplets. The three input channels containing aqueous solutions merge into a single channel which intersects a fourth perpendicular channel containing a water-immiscible carrier fluid. The same Poly(dimethylsiloxane) (PDMS) microfluidic chips as previously described (30) are used here. In short, SU8 (MicroChem) molds on silicon wafers are fabricated in a clean room. O_2_ plasma activation is employed to seal PDMS (Sylgard) replicas on a microscope slide. Flushing of 1% solution of 1H,1H,2H,2H-perfluorodecyltrichlorosilane (Alfa Aesar) in perfluorodecalin (PFD, Alfa Aesar) with subsequent rinsing with N_2_ ensures hydrophobization of the surface of the microfluidic channels. A 10:1 mixture of PFD with surfactant 1H,1H,2H,2H-perfluorooctanol (Alfa Aesar) serves as water-immiscible carrier fluid. The section of the square channel is 50 × 50 μm^2^. The microfluidic flow controller OB1 (Elveflow) is used for real-time, precise control and monitoring of pressures and flow rates in the 4 channels independently. The droplets propagation speed in the main channel is measured accurately as described in the Supplementary Information (See Supplementary Figure S1), and can be varied between 7 and 80 mm s^–1^. The water fraction, defined as the volume proportion of water in the main channel, was set to 0.7 by adjusting the oil flow with respect to the three aqueous flows. TRF measurements were then performed along the main microfluidic channel after a stable droplet flow was established.

Sub-picosecond 480-nm excitation pulses are produced from a Tangerine (Amplitude Systems) amplified fiber laser system with operating frequency of 50 kHz. An Optical Parametric Amplifier (OPA) developed in-house is pumped at 515 nm by the second harmonic of the fundamental Tangerine IR pulse. The OPA output pulse is then tuned and frequency doubled to set the excitation wavelength to 480 nm, with excitation power of about 30–50 μW. The 480-nm, pulsed laser beam is reflected by a dichroic mirror (495 nm edge dichroic beamsplitter, Semrock FF495-Di03-25 × 36) and focused in a 5-μm spot inside the main microfluidic channel to excite the Fl-labeled oligonucleotide (ON). The fluorescence light emitted by the droplets at the spot location is transmitted by the dichroic mirror and imaged through a spectrograph on the photocathode of a streak camera to perform time-resolved fluorescence measurements along the main microfluidic channel. A polarizer in front of the streak camera ensures magic angle (54.7°) relative orientation between detection and excitation polarization axes. Several fluorescence decay curves are accumulated from a large number of propagating droplets at different locations along the microfluidic channel after moving the microfluidic chip in front of the excitation spot. The streak camera temporal response function is almost a Gaussian function with a standard deviation of about 10 ps for the 1 ns detection time window. This standard deviation increases linearly with the selected detection time range. Therefore, the signal was successively registered in three time windows (1, 5 and 20 ns) and spectrally integrated. The final kinetic traces were reconstructed by appending the three data sets into one trace. By doing so, we combine the best possible time resolution enabled by the 1 ns time window and the accurate recording of the fluorescence decay tail until 20 ns. All of the registered fluorescence decay curves were assigned to a precise hybridization reaction time determined by knowing the distance from the T-junction (known by design of the microfluidic chip) and the droplets’ flow speed determined as described in detail in the SI (see Supplementary Figure S1). Importantly, the droplets’ speed was measured systematically during every individual mixing experiment.

### Chemicals

In this work, we used doubly-labeled (–)PBS_5′Dab_3′Fl (purchased from IBA GmbH, Germany)—with Dab and Fl standing for Dabcyl and 5(6)FAM, respectively - in order to monitor a fluorescence signal related to the end-to-end distance of the (–)PBS strand. Lyophilized ONs were dissolved in deionized water and their concentrations were determined from absorption spectra using the molar extinction coefficients at 260 nm provided by the supplier. The NC(11–55) peptide was synthesized using solid-phase peptide synthesis as previously described (36). Stock solutions of NC(11–55) were prepared with 2.5 equivalents of Zn^II^ in 25 mM Tris, 150 mM NaCl and 0.2 mM MgCl_2_ (pH 7.5). The protein concentration was determined from its absorbance at 280 nm using its extinction coefficient ϵ_280_ = 5700 M^–1^ cm^–1^. The structures of the PBS sequences, fluorescent labels, and NC(11–55) peptide are displayed in Scheme 1.

For all mixing experiments, the solutions of labelled (–)PBS and of the complementary, non-labelled (+)PBS (also purchased from IBA GmbH) were prepared separately—at various concentrations specified below for each experiment—in Tris buffer solution (25 mM Tris, 150 mM NaCl, 0.2 mM MgCl_2_, pH 7.5) and in the presence of NC(11–55) peptide added at a peptide:ON molar ratio of 1:1. NC(11–55) peptide was preferred to the wild-type NC(1–55) protein, because it causes less aggregation of nucleic acids at the concentrations used in this study (37). Moreover, all mixtures of PBS with NC(11–55) were prepared using prediluted solutions of both components in order to prevent local saturation and subsequent aggregation of PBS which must be avoided to investigate the biomolecular interactions under good conditions.

In order to analyze the data of the mixing experiments monitored by TRF in DmF chips, we also prepared separately the *equilibrated* extended duplex (+)PBS/ (–)PBS_5′Dab_3′Fl (ED) by mixing in a low binding Eppendorf tube the two complementary sequences at a 1.1:1 molar ratio of non-labeled to doubly-labeled ON. After annealing at 85°C for 3 min in a water bath, the mixture was slowly cooled down to room temperature. This equilibrated duplex was used to record reference fluorescence decay kinetics, as described in the results section below.

### Analysis of time-resolved fluorescence data

Fluorescence decay curves were fitted with multiexponential fitting functions. The decay kinetics are convolved by the instrument response function (IRF), which is correctly modeled by a Gaussian function with standard deviation σ. Therefore, we use the following fitting function:

(1) $$\begin{equation*}A\left( t \right) = {\rm{H}}\left( {t - {t_0}} \right)\mathop \sum \nolimits_i {A_i}*{e^{ - \frac{{\left( {t - {t_0}} \right)}}{{{\tau _i}}}}} \otimes \frac{1}{{\sigma \sqrt {2\pi } }}{e^{ - \frac{{{t^2}}}{{2{\sigma ^2}}}}},\end{equation*}$$

with *H*(*t*) the Heaviside step function (*H*(*t*) = 1 if *t*$ \ge$0, 0 if *t* < 0). The convolution ($ \otimes$) with the Gaussian IRF can be written analytically:

(2) $$\begin{eqnarray*} A\left( t \right) &=& C + \mathop \sum \nolimits_i \frac{{{A_i}}}{2}\exp \left( {\frac{{{\sigma ^2}}}{{2{\tau _i}^2}}} \right)\exp \left( { - \frac{{t - {t_0}}}{{{\tau _i}}}} \right) \nonumber \\ && \times\, \left[ {1 + {\rm{erf}}\left( {\frac{{t - {t_0} - {{{{\sigma ^2}}} \!\mathord{\left/ {\vphantom {{{\sigma ^2}} {{\tau _i}}}}\right.} \!{{{\tau _i}}}}}}{{\sigma \sqrt 2 }}} \right)} \right], \end{eqnarray*}$$

where erf is the error function which results from the convolution of *H*(*t*) by the Gaussian IRF. *C* is an offset accounting for the non-zero dark count rate of the streak camera.

The fits are performed by increasing the number of exponential components until the fit converges and the target parameters like time constants (${\tau _i}$) and corresponding amplitudes (${A_i}$), time origin (*t*_0_) and standard deviation $\sigma$ are determined. Convergence is achieved when the ‘weighted’ χ^2^ is minimized, i.e. as close to 1 as possible :

(3) $$\begin{equation*}{\chi ^2} = \mathop \sum \nolimits_i {\left( {\frac{{{y_i} - f\left( {{t_i}} \right)}}{{\sqrt {{y_i}} }}} \right)^2}\end{equation*}$$

meaning that the difference between experimental data points *y_i_* at time *t_i_* and the fitting trace *f*(*t_i_*) is minimum, considering the rms noise $\sqrt {{y_i}}$ of the Poisson statistics characterizing single photon detection experiments.

The outcome of the multiexponential fitting procedure is a set of amplitudes ${A_i}$ interpreted as the relative concentrations of the sub-populations characterized by the fluorescence decay times ${\tau _i}$, see details in the Results section below. The results of a TRF-DmF mixing experiment is thus the time evolution of the concentration of these sub-populations, inferred from the analysis of the TRF data recorded at several locations along the main microfluidic channel.

### Mixing experiments monitored with transient fluorescence intensity (Tr-FI) detection

In addition to transient TR-FRET performed with the TRF-DmF set-up, we also exploit transient fluorescence intensity (Tr-FI) to monitor the hybridization reaction when mixing 50 nM of (–)PBS_5′Dab_3′Fl with 1 μM to 5 μM of non-labeled (+)PBS in the same buffer solution as described above (25 mM Tris, 150 mM NaCl, 0.2 mM MgCl_2_, pH 7.5) and in the presence of NC (11–55) added at a 1:1 molar ratio to PBS. A stopped-flow apparatus (SFM-3, Bio-Logic) is used for Tr-FI monitoring of (–)PBS_5′Dab_3′Fl with excitation and emission wavelengths of 480 and 518 nm, respectively.

### Modeling of the hybridization kinetics

The results of all mixing experiments are quantitatively analyzed by fitting experimental data with the numerical solution of the rate equations of the following two-step model involving three sub-populations which are free (–)PBS, an intermediate (+)PBS/(–)PBS complex IC, and the final (+)PBS/(–)PBS extended duplex ED:


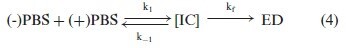


where *k*_1_ and *k*_–1_ are respectively, the association and dissociation rate constants of the intermediate complex, while *k_f_* is the extended duplex formation rate.

The sub-population kinetics obtained from several TRF-DmF mixing experiments were analyzed simultaneously in a global fit performed with a Python routine developed in-house using the lmfit package and implementing a numerical solution of the two-step model (Equation 4) as the fitting function. The known, total concentrations of (–)PBS and (+)PBS were fixed parameters specific to each mixing experiment, while the *k*_1_, *k*_–1_ and *k*_f_ rate constants were fitting parameters common to all mixing experiments and determined by a Levenberg-Marquardt least-squares minimization (38).

The fluorescence intensity decay kinetics obtained from several Tr-FI (stopped-flow) were analyzed by a global fit performed with the numerical solving software Dynafit (39,40). This program performs least-squares fits of the kinetic traces using the classical Levenberg-Marquardt algorithm. In this analysis, the fitting parameters common to all decay kinetics are the *k*_1_, *k*_–1_ and *k*_f_ rate constants and the fluorescence intensities of the various subpopulations.

## RESULTS

### Fluorescence decay kinetics of equilibrated SL (–)PBS and (+)/(–)PBS duplex in droplets

**Figure 2.**
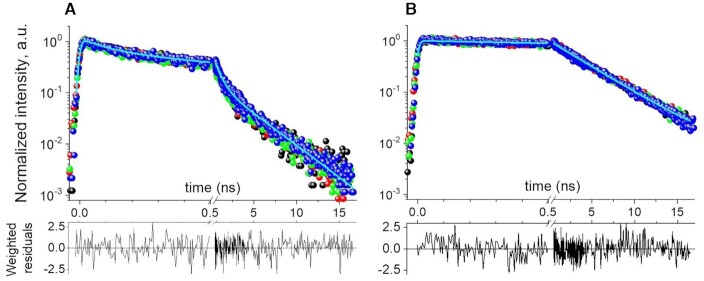
Fluorescence decay kinetics of 1 μM solutions of (**A**) stem-loop (–)PBS_5′Dab_3′Fl (SL) and (**B**) extended duplex (+)PBS/(–)PBS_5′Dab_3′Fl (ED), in the presence of 150 mM NaCl. Top panels: the measurements were done in the absence of NC(11–55) in cuvette (black) or droplets (green), and in the presence of NC(11–55) added at a peptide:ON molar ratio of 1:1 in cuvette (red) or in droplets (blue). The data acquired in microfluidic droplets in the presence of NC(11–55) (blue data) are fitted (cyan lines) to a triexponential (A) or monoexponential (B) function, and the corresponding residuals are shown in the bottom panels.

An important prerequisite to demonstrate the reliability of mixing experiments in microfluidic droplets is to make sure that the structures of the *equilibrated* stem-loop (SL) and extended duplex (ED) ONs are preserved inside the droplets, since adsorption at the water/oil interface may alter the structure of biomolecules (41). Hence in a preliminary experiment we checked that the fluorescence decay kinetics in microfluidic droplets for the FRET-labelled stem-loop(–)PBS_5′Dab_3′Fl (SL, before mixing) and for the extended duplex (+)PBS/(–)PBS_5′Dab_3′Fl (ED, after completion of the hybridization reaction) are the same as in spectroscopy cuvettes (i.e. bulk solution), as illustrated in Figure 2. To do so, we produced microfluidic droplets by injecting all three aqueous inlets of a microfluidic chip (see Figure 1A) with the same solutions of SL or ED, prepared as described in the Method section, but with various NaCl concentrations and with or without NC(11–55) added with a peptide:ON molar ratio of 1:1. Importantly, we observe (see Supplementary Figure S2) that the fluorescence decay kinetics of SL does depend on salt concentration inside the droplets, but not in spectroscopy cuvettes, illustrating the effect of the droplet water-oil interface at lower salt concentrations. Only at NaCl concentration as large as 150 mM, we observe identical decay kinetics in both droplets and cuvette (see Figure 2), indicating that the ON structures are identical in droplets and bulk solution. Hence, we use [NaCl] = 150 mM in all following experiments. We also checked that the fluorescence decay kinetics of SL and ED inside droplets remain invariant when measured at different locations along the main microfluidic channel (see supplementary Figure S3), i.e. after various propagation times from the T-junction until the chip outlet. We conclude that the initial SL hairpin structure and final ED structure are not perturbed inside the microfluidics droplets in the conditions described in the Methods section, as compared to bulk solution.

Quantitative analysis of the data displayed in Figure 2 reveals that the fluorescence decay kinetics for the *equilibrated* SL or ED samples are correctly fitted by a triexponential decay ${F_{SL}}( t ) = \mathop \sum \limits_{i = 1,3} {A_i}{e^{ - t/{\tau _i}}}$, with $\mathop \sum \limits_{i = 1,3} {A_i} = 1$, and a monoexponential decay ${F_{ED}}( t ) = {e^{ - t/{\tau _3}}}$, respectively, with amplitudes and time constants given in Table 1. These decay kinetics characterize the structures of the FRET-labeled (–)PBS before mixing and after completion of the hybridization reaction, respectively. The triexponential ${F_{SL}}( t )$ decay kinetics reveals a distribution of end-to-end distances indicating various degrees of opening of the SL hairpin stem, as previously described (33,42,43). At equilibrium, these structures interconvert on the sub-millisecond time scale, (43–47), i.e. much faster than the hybridization reaction progression, as we will see below. Therefore, we postulate that at any time along the hybridization process, residual free SL (–)PBS_5′Dab_3′Fl is always characterized by the same triexponential decay ${F_{SL}}( t )$ as determined here in the absence of the complementary (+)PBS strand. Importantly, Figure 2 also shows that the ${F_{SL}}( t )$ decay kinetics is not altered by NC(11–55) added at a peptide:ON molar ratio of 1:1. This absence of change in the decay kinetics is not the consequence of absence or partial binding since titration experiments (see SI, Supplementary Figure S4 and Table S1) reveal a ∼1 μM binding affinity of NC(11–55) for (–)PBS. Moreover, the preferential binding of NC(11–55) to the loop (48) explains that the stem and therefore the end-to-end distance distribution are not altered at this peptide:PBS molar ratio.

**Table 1.** Time-resolved fluorescence parameters of the equilibrated (–)PBS_5′Dab_3′Fl (SL) and (+)/(–)PBS_5′Dab_3′Fl (ED), in cuvette or droplets and in the presence or absence of NC(11–55) at a 1:1 peptide:ON molar ratio. The amplitudes A_i_ and lifetimes τ_i_ were obtained from the global (simultaneous) fit of all four curves shown in Figure 2A or B, with global reduced ${\chi ^2}$ of 1.07 and 1.03, respectively. The uncertainties represent the dispersion observed for each fitting parameter when fitting individually each curve of Figure 2A and B.

	(–)PBS_5′Dab_3′Fl	(+)/(–)PBS_5′Dab_3′Fl
	SL	ED
**A_1_**	0.46 ± 0.02	-
**τ_1_**	0.11 ± 0.02 ns	
**A_2_**	0.44 ± 0.01	-
**τ_2_**	0.71 ± 0.10 ns	
**A_3_**	0.10 ± 0.03	1
**τ_3_**	2.66 ± 0.25 ns	4.19 ± 0.12 ns
**τ_av_**	0.63 ns	4.19 ns
**QY^(a)^**	0.10	0.56

^(a)^See SI. We also measure QY^ref^= 0.57 for singly-labeled (+)PBS-Fl. The QY's are given with a standard deviation of 10%.

### Mixing experiments with the TRF-DmF set-up

Next, we record the evolution of the fluorescence decay curves of pre-steady-state complexes - produced upon droplet formation - during their propagation along the chip. These experiments are performed to reveal the kinetics of the structural relaxation and the population of transient states between the initial (ss) and final (ED) states, respectively characterized by ${F_{SL}}( t )$ and ${F_{ED}}( t )$ decay kinetics. For each mixing experiment, two solutions of (–)PBS_5′Dab_3′Fl and (+)PBS were prepared separately with NC(11–55) at a 1:1 peptide:ON molar ratio, and injected in two of the three aqueous inlets of the microfluidic chip. Tris buffer without NC(11–55) was injected in the third aqueous inlet (see Figure 1A). For each mixing experiment, typically 10 fluorescence decay kinetics are recorded successively at several locations along the microfluidic channel. This is done in about 3–4 h and consumes about 0.5 ml of each solution flowed through the microfluidic chip at a total flow rate ranging from 2.1 to 3.4 μl/min depending on the experiments.

**Figure 3.**
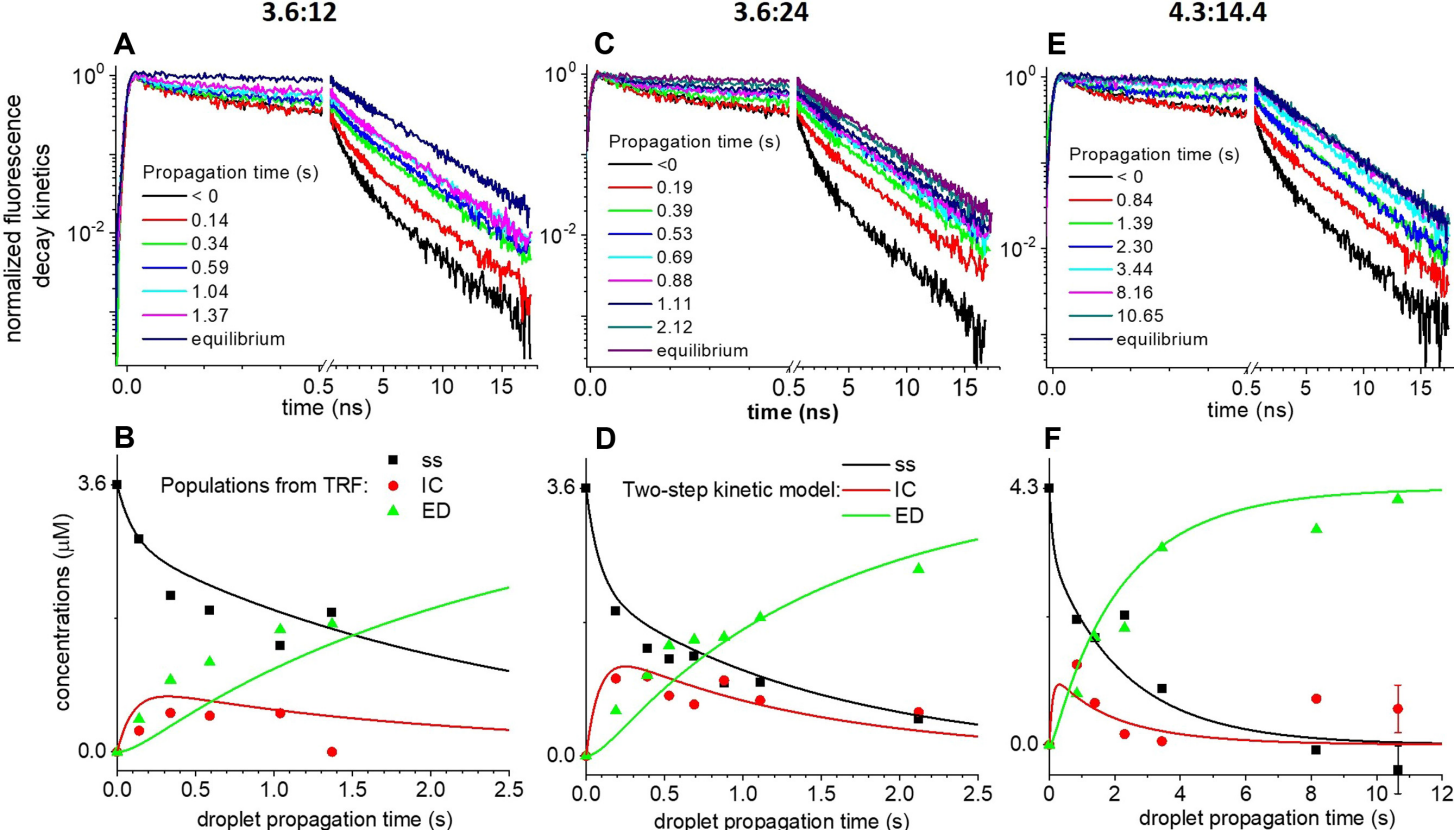
Evolution of the fluorescence decay kinetics (**A, C, E**) and corresponding species concentrations (**B, D, F**) as a function of droplet propagation time in the microfluidic chip. Solutions of ONs with NC(11–55) added at a 1:1 molar ratio are mixed in the ‘T’-junction. Total concentrations of (–)PBS:(+)PBS in μM are (A, B) 3.6:12, (C, D) 3.6:24, (E, F) 4.3:14.4 inside the droplets. Panels (A, C, E) display the fluorescence decay kinetics recorded in the microfluidic chip as a function of droplet propagation time. Negative (‘<0’) propagation time and ‘equilibrium’ refer to the fluorescence decay kinetics recorded in the microfluidic chip for the equilibrated (–)PBS and ED, respectively. Panels (B, D, F) display the results of the global fits of the fluorescence decay kinetics by Equation (6) which directly yield the proportions $1 - \alpha ( T ) - \beta ( T )$, $\beta ( T )$, and $\alpha ( T )$ of SL (squares), IC (dots) and ED (triangles) species, respectively, rescaled in units of concentrations knowing the total (–)PBS concentration (in μM). The time evolution of the populations are fitted (solid lines) assuming a two-step model (Equation 4). Buffer: 25 mM Tris, 150 mM NaCl, 0.2 mM MgCl_2_, pH 7.5.

Figure 3 displays the results and global analysis of three distinct mixing experiments where the total concentrations of (–)PBS_5′Dab_3′Fl and (+)PBS inside the droplets were 3.6 and 12, 3.6 and 24, 4.3 and 14.4 μM, respectively, and the concentration of NC(11–55) was equal to the total ON concentration (i.e. 15.6, 27.6, 18.7 μM, respectively). Panels A, C and E of Figure 3 display three collections of fluorescence decay kinetics recorded in each experiment, as a function of the droplet propagation time $T$ (in seconds) after the initial droplet formation and mixing event at the T-junction. The ‘negative’ propagation times refer to the decay kinetics recorded in the input channel—i.e. before mixing—for SL. They overlap the reference SL decay kinetics of Figure 2A. The ‘equilibrium’ decay kinetics is similar to that of (ED), recorded separately and displayed in Figure 2B.

To analyze the results of the mixing experiments, we use the previously determined decay kinetics ${F_{SL}}( t )$ and ${F_{ED}}( t )$ which characterize free SL and ED respectively. As a preliminary analysis, we try to fit globally all decay kinetics of all three experiments to a first fitting function written as:

(5) $$\begin{equation*}{F_1}\left( {t,T} \right) = \left( {1 - \alpha \left( T \right)} \right){F_{SL}}\left( t \right) + \alpha \left( T \right){F_{ED}}\left( t \right),\end{equation*}$$

where $T$ is the droplet propagation time (in seconds) corresponding to the ‘slow’ hybridization reaction kinetics, while *t* (in ns) corresponds to the ‘fast’ fluorescence decay kinetics recorded at each $T$ value, and $\alpha ( T )$—the only fitting parameter for each curve—describes the progress of a 1-step reaction converting free SL directly into ED. When doing so, the residuals of the fit evidence a very short fluorescence decay component (<0.1 ns), which is systematically not captured by this fitting function in all three experiments for the early propagation times $T$ (Supplementary Figure S5 in SI). This preliminary analysis thus evidences a very short decay component that is transiently observed upon mixing both complementary strands, and that is not observed in the equilibrated SL or ED species. We interpret this short fluorescence decay component as the evidence and signature for an intermediate complex (IC). We therefore perform a second global analysis of all data, with a second fitting function:

(6) $$\begin{eqnarray*} {F_2}\left( {t,T} \right) &=& \left( {1 - \alpha \left( T \right) - \beta \left( T \right)} \right){F_{SL}}\left( t \right) + \beta \left( T \right){F_{IC}}\left( t \right) \nonumber \\ && +\, \alpha \left( T \right){F_{ED}}\left( t \right) \end{eqnarray*}$$

We postulate a monoexponential decay ${F_{IC}}( t ) = {e^{ - t/{\tau _{IC}}}}$ with a unique time constant ${\tau _{IC}}$ common for all decay kinetics in all three experiments, and globally fit all fluorescence decay kinetics with $\alpha ( T )$ and $\beta ( T )$as additional fitting parameters for each curve. This results in a very good global fit (Supplementary Figure S6 in SI), with an improved reduced χ^2^ value of 1.05 instead of 1.14 for the preliminary analysis with Equation (5). We note that χ^2^ = 1.05 is about the most accurate fit we can achieve given the present signal-to-noise ratio and experimental reproducibility illustrated in Figure 2 and Table 1. The global fit reveals ${\tau _{IC}} = \;$0.045 ns, assigned to the IC. The results of the fit to Equation (6) are displayed by the points in panels B, D and F of Figure 3, which represent the amplitudes $1 - \alpha ( T ) - \beta ( T )$, $\beta ( T ),$ and $\alpha ( T )$, identified with the concentrations of SL, IC and ED, respectively, and rescaled in units of concentrations knowing the total concentration of (–)PBS in the microfluidic droplets in each experiment. We evaluate the relative uncertainty on the concentrations of the three species to be 5–10% in the final analysis (Figure 3B, D, F). Accordingly, two representative errors bars are displayed for the last time point in Figure 3F.

Finally, the time evolution of the concentrations of the three species is quantitatively analyzed using the 2-step hybridization reaction model described by Equation (4). We perform a global fit of the results of the three experiments (i.e. panels B, D and F of Figure 3) with the numerical solution for the rate equations for the second-order reaction kinetics described in Equation (4). The formation rate of IC appears to be in the range of *k*_1_ = 10^5^ M^–1^ s^–1^ or larger. Actually, given the dispersion (∼0.3–0.5 μM) on the species concentrations extracted from the above fluorescence decay analysis, any larger k_1_ value yields essentially the same minimal χ^2^ value. Values of *K* = *k*_1_/*k*_-1_ = (3.5 ± 1.5) x 10^4^ M^–1^ and *k*_f_ = 1.5 to 2 s^–1^ are obtained for the IC equilibrium constant and the ED formation rate, respectively.

### Complementary mixing experiments with Tr-FI detection

**Figure 4.**
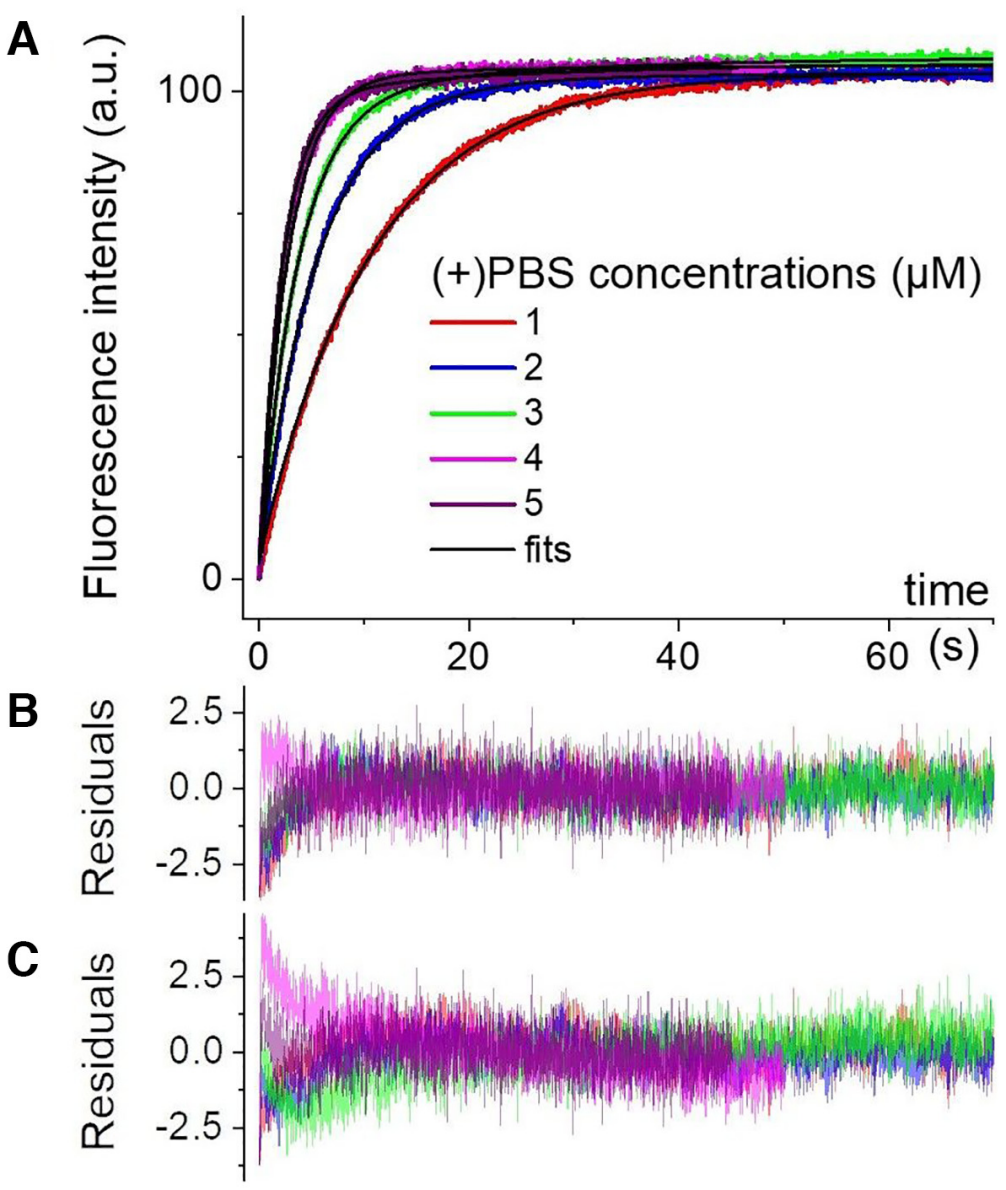
Transient fluorescence intensity (Tr-FI) kinetic traces of NC(11–55)-promoted (+)/(–)PBS annealing reaction. (**A**) Mixing of 0.05 μM (–)PBS_5′Dab_3′Fl with 1 μM (red), 2 μM (blue), 3 μM (green), 4 μM (magenta) and 5 μM (purple) (+)PBS in the presence of NC(11–55) at a 1:1 peptide:ON ratio. Fittings of the kinetic traces are shown as black lines. Residuals for the fits (Dynafit software, see Materials and Methods section) of the kinetic traces with (**B**) a two-step model—Equation (4)—and (**C**) a three-step model (see text). The rate constants obtained by both fits are given in Table 2.

Previous investigations of the (+)/(–)PBS hybridization reaction have been performed by Tr-FI (33–35,42) or gel electrophoresis (49) using different NC(11–55), salt or complementary strand concentrations. Hence to carefully assess the advantage of the TRF-DmF approach, we performed complementary experiments by mixing (–)PBS_5′Dab_3′Fl with (+)PBS in the same conditions as above, but monitored with Tr-FI. The results are displayed in Figure 4.

For the Tr-FI experiments performed with (–)PBS_5′Dab_3′Fl (Figure 4), we obtain *k*_1_ = 0.91 × 10^5^ M^–1^ s^–1^ in good agreement with the TRF-DmF data above. However, regarding the other two rate constants, the Dynafit routine converges with *k*_–1_ = 0.0012 s^–1^ and *k*_f_ = 0.045 s^–1^ with the additional information that IC and ED have similar fluorescence intensity (*I*_IC_ = 0.95 x *I*_ED_). These results are in striking contrast to those from the TRF-DmF experiments which (i) yield *k*_–1_ and *k*_f_ values two to three orders of magnitude faster and (ii) evidence IC as being essentially dark as compared to ED, with a fluorescence lifetime two orders of magnitude shorter (0.045 ns versus 4.2 ns). These differences could be rationalized by the fact that (i) the dark transient species evidenced by TRF certainly remains undetectable with fluorescence intensity detection and (ii) the bright transient species evidenced by the Tr-FI experiment converts into the final ED on a time scale (*k*_f_^–1^ ∼20 s) significantly slower than the reaction times monitored in the DmF experiments (up to ∼10 s, see Figure 3F). Altogether, we conclude that the ICs detected in both experiments must be two distinct species, namely IC1 a dark species detected only by Tr-TRF, and IC2 a species nearly as bright as ED and converting slowly into ED. These two distinct ICs also explain the very different *k*_–1_ and k_f_ rates as we will argue in the Discussion section below. It should be noted that the values of *k*_1_ and *k_f_* are highly reproducible in the fits of the Tr-FI kinetic traces. In contrast, the *k*_–1_ value is less reliable, likely because it is at least one order of magnitude lower than the k_f_ value and thus appears negligibly small.

## DISCUSSION

In this work, an original TR-FRET experiment performed in a DmF chip was used to monitor in real time the structural evolution of the hairpin-shaped doubly-labelled (–)PBS oligonucleotide during its annealing with its complementary (+)PBS sequence promoted by the nucleic acid chaperone NC(11–55) peptide. The (+)/(–)PBS system was selected because its annealing kinetics in the absence and presence of NC(11–55) or NC(1–55) has already been well characterized (33–35,42,49). In this system, the fluorescence lifetime signatures of the doubly-labelled (–)PBS_5′Dab_3′Fl in its SL and ED forms are markedly different. Indeed, SL is characterized by three lifetimes ranging from 0.11 ns to 2.66 ns, which reveal an equilibrium distribution of molecular structures achieving various end-to-end distances (43). In contrast, ED is characterized by a single 4.19 ns lifetime, corresponding to the unquenched Fl lifetime due to the large distance (>6 nm) between Fl and Dab in the ED. As the TRF-DmF experiments allow monitoring the lifetime distribution of the NC(11–55)-promoted (+)/(–)PBS annealing reaction over time, we could for the first time directly observe an IC associated with a 50-ps-short fluorescence lifetime, slightly shorter than the shortest (110 ps) of the three lifetimes of the (–)PBS_5′Dab_3′Fl SL, indicating that (–)PBS specifically adopts a very short end-to-end distance in this IC. Assuming a fast structural interconversion of the (–)PBS SL structures, (43) we propose that the entire SL population decays via this structure characterized by a very short end-to-end distance, which represents only a fraction of the (–)PBS SL population at equilibrium, but is probably the reactive species to produce the IC. In our experimental conditions, this IC accumulates within less than 1 s and then its concentration decreases with time.

Further information on the NC(11–55)-promoted annealing reaction was obtained by comparing the TRF-DmF data with Tr-FI data obtained by mixing (–)PBS_5′Dab_3′Fl with (+)PBS in the same conditions. Analysis of the Tr-FI kinetic traces using the same two-step model (Equation 4) as for TRF-DmF shows large differences in the *k*_–1_ and *k*_f_ values (Table 2) as well as in the IC brightness that strongly suggest the existence of two ICs. The much faster *k*_f_ rate (1.5 s^–1^) in the TRF-DmF experiment (versus 0.045 s^–1^ in Tr-FI) suggests that the low fluorescent IC1 observed in the TRF-DmF experiment is converted into a subsequent bright intermediate, IC2 observed in Tr-FI. Hence, we define *k*_2_ = 1.5 s^–1^ as the IC2 formation rate obtained from the TRF-DmF experiment and *k*_f_ = 0.045 s^–1^ as the actual ED formation rate obtained from the Tr-FI experiment only. Indeed, we argue that ED cannot be observed in TRF-DmF because (i) IC2 and ED must have nearly identical fluorescence lifetimes since they have similar brightness (*I*_IC2_ = 0.95 x *I*_ED_), and (ii) the conversion rate of IC2 is too slow to be observed within the reaction times accessible in the TRF-DmF experiment. With a rate constant of 1.5 s^–1^, IC2 formation is faster than IC1 formation (= *k*_1_[(+)PBS] < 0.1 s^–1^ in all Tr-FI kinetics), which explains why the growth of the IC2 signal detected in Tr-FI is limited by the formation speed of the non-detected IC1, and why both Tr-TRF and Tr-FI reveal a similar second-order IC formation rate *k*_1_.

**Table 2.** Summary of the kinetic parameters corresponding to the fits of the TRF-DmF and Tr-FI data with the 2-step and 3-step models

Experiment	Model	*k*_1_, 10^4^ (M^–1^s^–1^)	*k*_–1_ (s^–1^)	*k*_2_ (s^–1^)	*k*_–2_, 10^–3^ (s^–1^)	*k*_f_, 10^–2^ (s^–1^)
Tr-TRF	Two-step	≥10	4 ± 2	2 ± 0.5	-	-
Tr-FI	Two-step	9.1 ± 0.1	-	-	1.2 ± 0.2	4.5 ± 0.1
Tr-FI	Three-step	28 ± 1	6.2 ± 0.3	3.8 ± 0.5	<1	6.7 ± 0.5

**Scheme 2.**
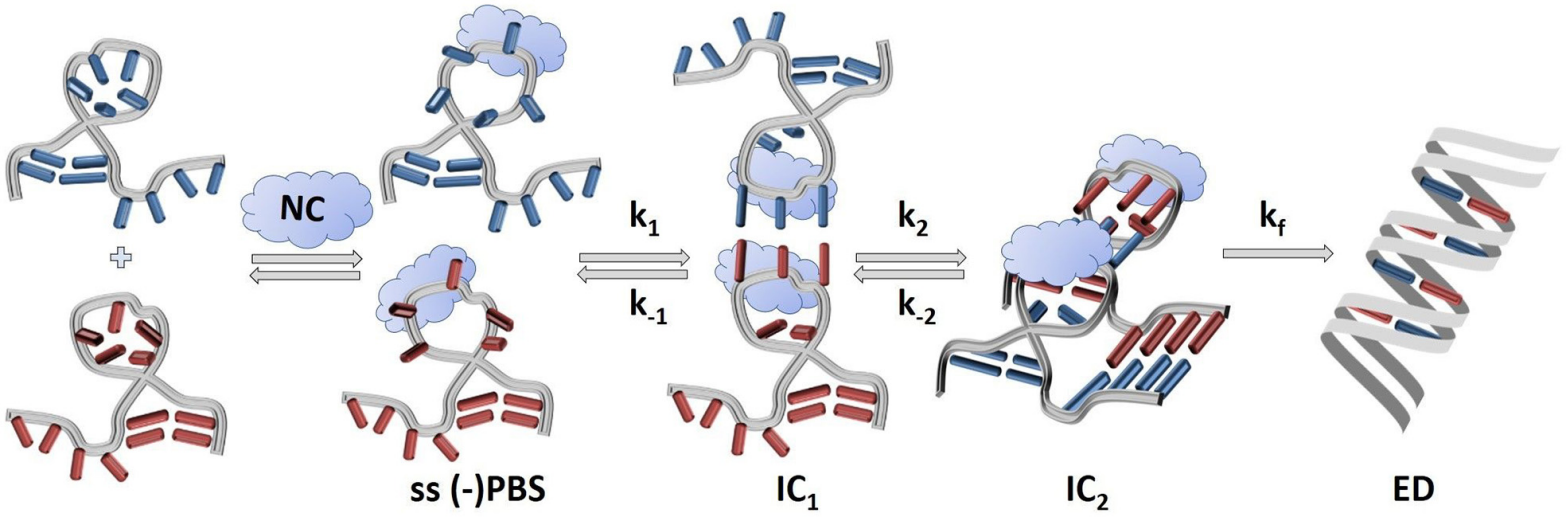
Three-step reaction model for the (+)/(–)PBS annealing in the presence of NC(11–55) at a 1:1 peptide:ON molar ratio.

Taken together the above results lead us to propose the 3-step model depicted in Scheme 2 for the NC(11–55)-promoted (+)/(–)PBS hybridization kinetics. Because (–)PBS is in a closed structure (very efficient FRET) in IC1, and because the NC(11–55) protein was shown to specifically bind to the loop when added at a 1:1 molar ratio, (48) we identify the NC(11–55)-promoted IC1 to a so-called ‘loop-loop kissing complex’ the formation of which is the limiting step. When produced, this complex then rearranges into the more stable IC2 complex characterized by a significantly larger end-to-end distance, suggesting further hybridization of the stem part and overhangs, until further rearrangement into the final ED on a much slower time scale.

To further validate the consistency of our model, we fitted the Tr-FI kinetic traces with the Dynafit software using the complete three-step model of Scheme 2 (Figure 4С). As the lifetime of IC1 (45 ps) is 100-fold smaller than the lifetime (4.19 ns) of ED, we fixed its brightness *I*_IC1_ = 0.01 x *I*_ED_ as the only constraint. The obtained *k*_–1_ and *k*_2_ values are in excellent agreement with the corresponding values deduced from the TRF-DmF data fitted with the two step model, while the *k*_–2_ and *k*_f_ values were in full agreement with the corresponding values for the Tr-FI data fitted with the two-step model (Table 2). Moreover, the *k*_1_ value is consistent with the lower boundary value determined with the TRF-DmF data. As expected, the product *k*_1_ × *k*_2_/*k*_–2_ = 1.7 × 10^5^ M^–1^ s^–1^ calculated from the three-step model fit of the Tr-FI data matches well with the *k*_1_ value (0.95 × 10^5^ M^–1^ s^–1^) obtained from the two-step model fit. A perfect match between the three-step model and the two-step model is also found for the IC2 fluorescence intensity that is observed to correspond to 94% of that of ED. Finally, the three-step model fit confirms that the *k*_–2_ value is negligibly small as compared to the *k*_f_ value, suggesting that the conversion of IC2 to ED is nearly irreversible. This is consistent with our model where extensive base-pairing between the loops and overhangs may lead to up to nine base pairs (five in the loops and four in the overhangs) in IC2, which makes its dissociation unlikely.

## CONCLUSION

This work demonstrates the strength of the proposed TRF-DmF approach to investigate the kinetics of biologically-relevant NA structural rearrangements. The production of water-in-oil microreactors enables rapid mixing (millisecond timescale) to create pre-steady-state conditions necessary to monitor irreversible (bio)chemical reaction kinetics. The few-hundred picolitre microreactors can also be accurately manipulated in microfluidic chips. Here, we simply propagate them at controlled speed over known, centimeter-scale distances to enable their monitoring at well-controlled time delays—from milliseconds to minutes—after the initial mixing event. Then, implementing TRF detection along the microfluidic channel for TR-FRET sensing enables monitoring a distribution of biomolecular structures as a function of the microreactors propagation time. More precisely, the analysis of the TRF decay kinetics in terms of a distribution of fluorescence lifetimes (${\tau _i}$) and corresponding amplitudes (${A_i}$) provides structural information (since ${\tau _i}$ encodes the donor-acceptor relative distance and orientation) as well as the relative concentration of the corresponding species, respectively.

In contrast, the simultaneous monitoring of the concentrations of the various species involved is clearly impossible with Tr-FI based techniques, which provide an integrated signal of all species in solution and do not allow to directly monitor transient species that cannot be isolated. Only indirect information can be obtained on IC through fits of the kinetic traces to a given reaction model. Finally, the present case of a nearly dark IC which negligibly contributes to the total intensity (<1%) remains unnoticed in Tr-FI experiments, which explains that it has never been reported so far, and illustrates the unprecedented sensitivity achieved by the TRF-DmF approach.

Molecular reaction kinetics are also frequently characterized by single molecule (SM) FRET experiments. When performed under equilibrium conditions, such experiments are not suited for the systems described in this paper. However, microfluidics devices have also been used to investigate irreversible (bio)chemical reaction kinetics by SM experiments in pre-steady-state conditions, using confocal or widefield SM detection schemes. In the confocal scheme (50,51) single molecules diffuse freely through the detection volume, which requires a strong dilution to reach the SM level. In these conditions, complexes displaying moderate or low affinity such as those investigated in our study cannot be investigated. Alternatively, the widefield geometry (52,53) requires to functionalize and immobilize one partner on the glass surface. Although, this enables improving the measurement statistics thanks to a parallelized detection scheme, it does not allow a precise control of concentration and the photobleaching of the dyes limits the total observation time.

Comparatively, the microdroplet technology offers the advantage of an accurate control of concentration with no upper limit except for molecule aggregation, and no photobleaching issues due to the very short exposure time ($ \le$1 ms) of individual droplets travelling in front of the laser focus. A well-known drawback is the possible, non-specific adsorption of biomolecules at the water-oil interface which presumably perturbs the system of interest like immobilization does in widefield SM experiments. However, appropriate surfactants have been successfully designed to mitigate this problem, (41,54) and TRF is the ideal approach to identify residual alteration of the biomolecule structures—as we also showed above—thus enabling further control and optimization of the experimental conditions.

In the present implementation of TRF-DmF, we used a streak camera offering a sub-10-ps time resolution required to evidence the 50-ps fluorescence decay time characterizing the early IC. When enough excitation laser power is available, a streak camera also enables parallelizing data acquisition by engineering light-sheet illumination of the microfluidic chip so as to monitor simultaneously the fluorescence decay kinetics at several locations along the chip, as we originally demonstrated (30). Here, due to limited laser power at 480 nm, we rather used single spot excitation. Hence a single-channel detection scheme is enough which may be implemented using a single-photon avalanche diode (SPAD) and time-correlated single photon counting (TCSPC) electronics. This would possibly enable a better signal-to-noise ratio (lower dark count rate as compared to the photocathode of the streak camera), but degrade the time-resolution to about 50 ps. Still it remains much better than the 1-ns time resolution offered by the so-called ‘direct waveform recording’ technique (55) used in previous implementations of *transient* Time-Resolved FRET (22).

While implementations of TRF-DmF using TCSPC were already demonstrated—by us and others (56)—to be relevant for innovative high-throughput screening (57,58) or droplet sorting (59) applications, the present work explores a second, qualitatively different field of application of TRF-DmF. Indeed, our data suggest that in combination with an ever-growing set of innovative fluorescent probes, TRF-DmF will offer unprecedented opportunities and expectations towards in-depth—rather than high-throughput—investigations of biomolecular reaction kinetics from the ms time scale to minutes, with very low material consumption and exquisite sensitivity at evidencing and characterizing transient biomolecular structures. Applications in the field of NAs are particularly promising because they can be easily fluorescently labeled and numerous protein- or ligand-induced NA interconversions are nearly irreversible, so that the pre-steady-state conditions offered by TRF-DmF technique is particularly suited for their investigation.

## DATA AVAILABILITY

All data supporting the conclusions of this work are disclosed in the paper and Supplementary Information file and are available upon request.

## Supplementary Material

gkab687_Supplemental_File
