# Supplementary material for: Kinetics of protein-assisted nucleic acid interconversion monitored by transient time resolved fluorescence in microfluidic droplets
Source: Nucleic Acids Res. 2021 Aug 27;49(19):e111. doi: 10.1093/nar/gkab687 (PMC8565319; doi:10.1093/nar/gkab687)
Supplement: gkab687_Supplemental_File [file gkab687_supplemental_file.pdf]

Supplementary Information for

Kinetics of Protein-assisted nucleic acid interconversion monitored by transient time resolved fluorescence in droplet microfluidics

Natalia Grytsyk,[a),b)] Damien Cianfarani,[a)] Olivier Crégut,[a)] Ludovic Richert, [b)] Christian Boudier [b)], Nicolas Humbert [b)], Pascal Didier [b)], Yves Mély,*[b)] Jérémie Léonard.*[a)]

[a)] Institut de Physique et Chimie des Matériaux de Strasbourg, Université de Strasbourg & CNRS, 67034 Strasbourg, France.

[b)] Laboratoire de Bioimagerie et Pathologies, UMR 7021 CNRS, Université de Strasbourg, Faculté de Pharmacie, 67401 Illkirch, France.

[*)] corresponding authors: yves.mely@unistra.fr, Jeremie.Leonard@ipcms.unistra.fr

List of abbreviations:

FRET = Förster Resonant Energy Transfer
TRF = time-resolved Fluorescence
DmF = droplet microfluidics
TRF-DmF = the experimental set-up implementing TRF detection of microdroplets in a microfluidic chip
NA = nucleic acid
SM = single molecule
SM-FRET = single-molecule FRET
TR = time-resolved
TR-FRET = time-resolved FRET
TRF = time-resolved fluorescence
FI = Fluorescence intensity
Tr-FI = Transient fluorescence Intensity (i.e. conventional stopped-flow with FI detection)
SL = stem loop (-)PBS
ED = hybridized (+)/(-)PBS extended duplex
IC = intermediate complex
ON = oligonucleotide

Measurements of the droplets' flow speed

In order to determine the relation between the propagation distance of the droplets and the reaction time, it is important to measure the flow speed of the droplets. It can be calculated as the ratio of the total flow of the injected fluids to the section of the channels. However, the problem is that the height of the channels, fixed by the height of the SU8 resin on the mold (photolithography step), is not always known precisely. In fact, the thickness of the resin can fluctuate by several micrometers because of the production method. In addition, due to the pressure drop along the channel and to the elasticity of PDMS, the channel section continuously reduces from the input where high pressure is applied to the output at ambient pressure. Since the flow rate is certainly constant, the droplet speed continuously increases (can increase by up to 44 %) along propagation.

One way of measuring the speed is to measure the distance propagated by the droplets during a known interval of time.[1] To do so, a CCD camera (Hamamatsu C8484) is used to record micrographs of the chip. We associate a function generator (DS340, Standford Research Systems) with a LED panel to

produce a pair of light pulses separated by a known time interval Δt during the CCD camera exposure time. Thus, it is possible to observe the images of double droplets (Fig. S1-A), each being separated by a certain distance that we measure. Nevertheless, the measured distance is very small and therefore the error of the calculated flow speed will be large. A way to improve precision of the measurements is to find larger intervals Δt when the droplets are not doubled anymore but are precisely superimposed like in the Fig. S1-B. In this case we measure larger distances with smaller errors.

(A)

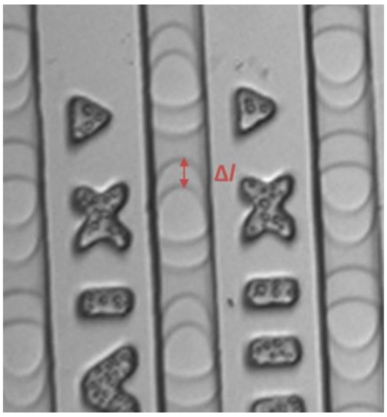

(B)

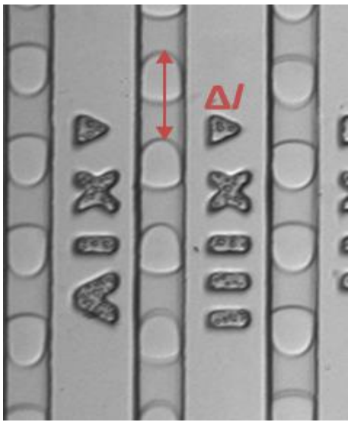

Figure S1. Flow speed measurement. (A) Typical image used to measure flow speed of the droplets. The droplets propagate a distance Δl during the time Δt between two short light pulses. (B) An image used to measure flow speed of the droplets with higher accuracy. In this example, the droplet flow speed is measured to 34.4 ±0.2 mm s^{-1}

Influence of NaCl concentration on the fluorescence decay kinetics measured in droplets

We observe that the decay kinetics of SL (-)PBS_5’Dab_3’Fl depends on the NaCl concentration inside microfluidic droplets but not in conventional spectroscopy cuvette, as illustrated in Figure S2. The same solutions are measured either in a cuvette (Figure S2-A) or inside droplets upon injection of the solutions in the three aqueous inlets of a microfluidic chip (Figure S2-B). Figure S2-B suggests that at lower NaCl concentration, the structure of SL (-)PBS_5’Dab_3’Fl in droplets is altered, probably due to adsorption at the water/oil interface. The cuvette and droplet data become identical for 150 mM NaCl concentration. We propose that higher ionic strength prevents adsorption at the water/oil interface or enhances solvation of oligonucleotides in the droplets. Considering these data, all subsequent experiments were performed using oligonucleotides dissolved in a buffer with 150 mM NaCl.

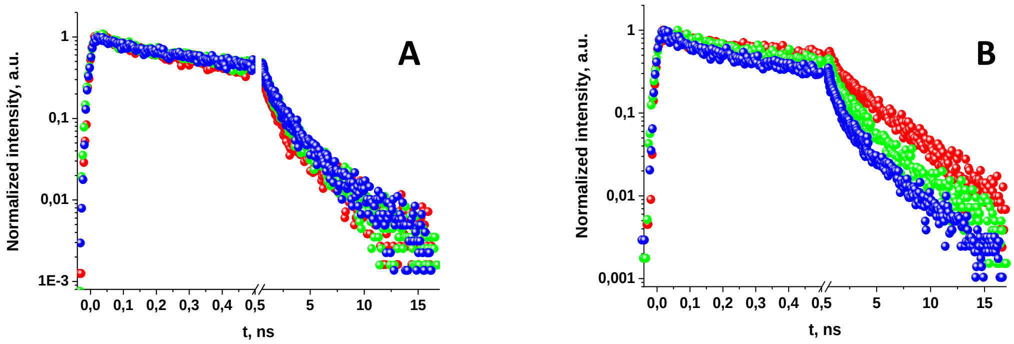

Figure S2. Fluorescence decay curves of SL (-)PBS_5'Dab_3'Fl in Tris-HCl buffer without NC (11-55) as a function of NaCl concentration in (A) conventional spectroscopy cuvettes and (B) microfluidic droplets circulating in the microfluidic chip. Salt concentrations are 30 mM (red), 100 mM (green) and 150 mM (blue). The decay kinetics in cuvette and droplets are identical for [NaCl]=150 mM (blue curves in panels A and B), as shown in Figure 2 of the main paper.

Measurements at different positions in the chip

In order to demonstrate that inside the droplets, the conformations of the equilibrated SL (-)PBS_5'Dab_3'Fl and (+)/(-)PBS_5'Dab_3'Fl duplex in the presence of 1 equivalent of NC (11-55) are stable during their propagation in the microfluidic chip, we performed reference measurements at different locations along the main microfluidic channel downstream the T-junction. The results are shown in Figure S3.

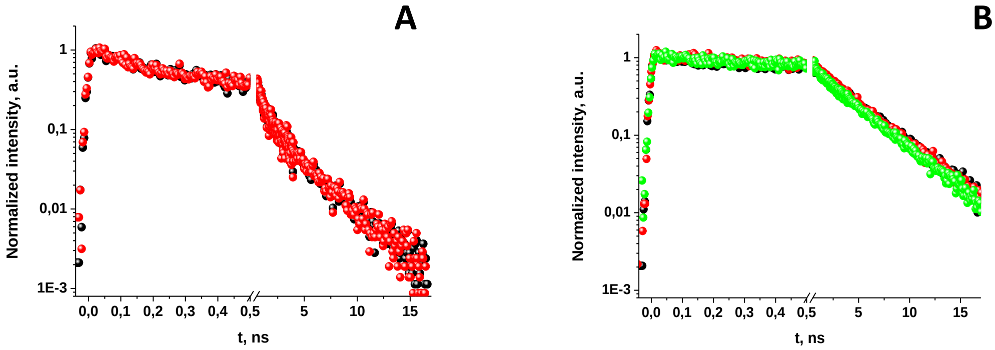

Figure S3. Invariance of the fluorescence decay kinetics with droplets propagation inside the main microfluidic channel. (A) Fluorescence decays of SL (-)PBS_5'Dab_3'Fl measured in droplets after 2.7 mm (black) and 16.1 mm (red) of propagation in the main channel after T-junction. (B) Fluorescence decays of ds (+)/(-)PBS_5'Dab_3'Fl in droplets after 2.7 mm (black), 16.1 mm (red) and 30.8 mm (green) of propagation in the main channel after the T junction, corresponding to 0.27, 1.61 and 3.08 s propagation times at a droplet flow speed of 10 mm.s^{-1} here.

NC binding constant to (+)PBS: titration experiments

To determine the percentage of free and bound PBS forms in the presence of NC(11-55) in microfluidic experiments, binding experiments are performed in the same buffer as in the microfluidics experiments. Binding of NC(11-55) to (+)PBS labeled with fluorescein probe ((+)PBS_Fl) is monitored by fluorescence anisotropy. By titrating (+)PBS_Fl with increasing concentrations of NC(11-55), we obtain titration curves as illustrated in Figure S4.

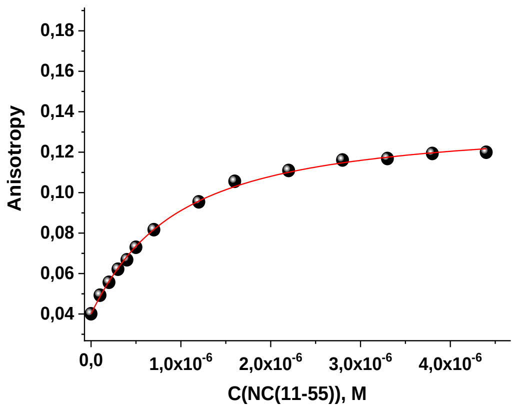

Figure S4. Titration of 100 nM (+)PBS_Fl with increasing concentrations of NC(11-55). Excitation and emission wavelengths are 480 nm and 520 nm, respectively. Measurements are performed at 20°C in 25 mM Tris, 150 mM NaCl, 0.2 mM $MgCl_2$ (pH 7.5). Experimental points are fitted using equation S1 (red line).

Titrations performed at 100 nM (Figure S4) and 300 nM (not shown) (+)PBS_Fl are fitted with equation S1 in order to determine the binding affinity (K):

$$S = S_0 + (S_t - S_0)Y \text{ with } \mathrm{Y} = \left(\frac{1+(\mathrm{P}_t+\mathrm{nN}_t)K-\sqrt{(1+(\mathrm{P}_t+\mathrm{nN}_t)K)^2-4\mathrm{P}_t n \mathrm{N}_t K^2}}{2K\mathrm{N}_t}\right) \qquad \text{(S1)}$$

where S_0 and S_t are the initial and final anisotropies, respectively; P_t, the concentration of protein (M); n, the stoichiometry (n=1) and N_t, the concentration of (+)PBS_Fl (M).

A dissociation constant K_d = 1/K of 1.3 ± 0.4 µM is obtained from the titrations.
This allows us to calculate the percentage of NC(11-55)-bound PBS under our experimental conditions using:

$$\alpha_{PBS\_NC} = \frac{K[NC]}{1 + K[NC]}$$

where $[NC]$ is the concentration of NC(11-55). Results are summarized in the Table S1.

Table S1. Calculated populations (α_{PBS_NC}) of PBS bound with NC(11-55) at the experimental conditions used in Figures 2 and 3.

**(-)PBS (µM) : (+)PBS (µM)**	**Total concentration of NC(11-55), µM**	α_{PBS_NC}, %
1 : 1.1	2.1	64 ± 2
3.6 : 12	15.6	92 ± 2
3.6 : 24	27.6	95 ± 3
4.3 : 14.4	18.7	93 ± 2

These results indicate that in the mixing experiments in droplets (Figure 3), PBS exists predominantly (≥92%) in bound form with NC(11-55).
As reported previously,[2] the PBS site with the highest affinity for NC(11-55) is located in the loop. Thus, as NC(11-55) is added to PBS at a 1:1 molar ratio, it will predominantly bind to PBS loop and thus, not destabilize or change the structure of the PBS stem.

Determination of the fluorescence quantum yields and population of “dark” species

The fluorescence quantum yields (QY) of SL (-)PBS_5’Dab_3’Fl and (+)/(-)PBS_5’Dab_3’FI duplex were determined using fluorescein in 0.1 M NaOH as a reference. Calculations of the QY, that is the ratio of the number of emitted photons to the number of the absorbed ones, were performed according to:

$$QY_x = QY_r \frac{I_x}{I_r}\frac{A_r}{A_x}\left(\frac{n_x}{n_r}\right)^2 \qquad \text{(S2)}$$

where QY_x is the fluorescence quantum yield of the sample, QY_r is that of the reference dye, I_x and I_r are their corresponding integral fluorescence intensities, A_x and A_r are the respective absorbance of the sample and the reference at the excitation wavelength, n_x and n_r are the refractive indices of the corresponding solvents of the sample and reference.

The major source of uncertainty in the determination of QY of the SL (-)PBS_5’Dab_3’Fl sample is the measurement of the absorbance of its fluorescein probe at the excitation wavelength. The difficulty in this measurement is that the absorbance of fluorescein (range 400 to 550 nm) overlaps with the absorbance of Dabcyl (range 350 to 575 nm). Therefore, in order to determine the absorbance of fluorescein without contribution from Dabcyl we deconvoluted the absorption spectrum of SL (-)PBS_5’Dab_3’Fl. Deconvolution was done without taking into account the probable excitonic interaction of two probes that likely shifts their absorbance spectra.[3] Therefore, the determined absorbance value of the fluorescein probe from the deconvoluted spectrum and thus, the quantum yield of SL (-)PBS_5’Dab_3’Fl have an uncertainty of at least 10%.

Obtained values of quantum yields allow us to calculate the population of the so called “dark” species and check whether they are present in our system. These non-emissive “dark” species would actually correspond to species having fluorescence lifetimes shorter than the time resolution of the streak camera due to either static or fast dynamic quenching. The population of “dark” species was calculated according to the following formula:

$$\alpha_0 = 1 - \frac{QY\,\langle\tau\rangle^{ref}}{\langle\tau\rangle\,QY^{ref}} \qquad \text{(S3)}$$

where $\langle\tau\rangle^{ref}$ and $\langle\tau\rangle$ are the mean lifetimes of the reference (singly labelled (+)PBS_Fl) and the sample (SL (-)PBS_5’Dab_3’Fl or ds (+)/(-)PBS_5’Dab_3’FI), respectively; QY^{ref} and QY are their corresponding quantum yields.

Our calculations indicate that $\alpha_0 = 0$ in either SL (-)PBS or (+)/(-)PBS ED, so that all the conformations of SL (-)PBS_5’Dab_3’Fl can be monitored by time-resolved measurements upon formation of the ED. This behavior is in sharp contrast to that previously observed on (-)PBS doubly labelled by 6-carboxyrhodamine (Rh6G) at its 5’ terminus and by Dab at its 3’ terminus, where “dark” non-emissive species represented 76% [4]. This difference might be explained by the quenching that results from the electron transfer between excited Rh6G and guanine residues.[5]

Global fit of the decay kinetics recorded in three microfluidics experiments

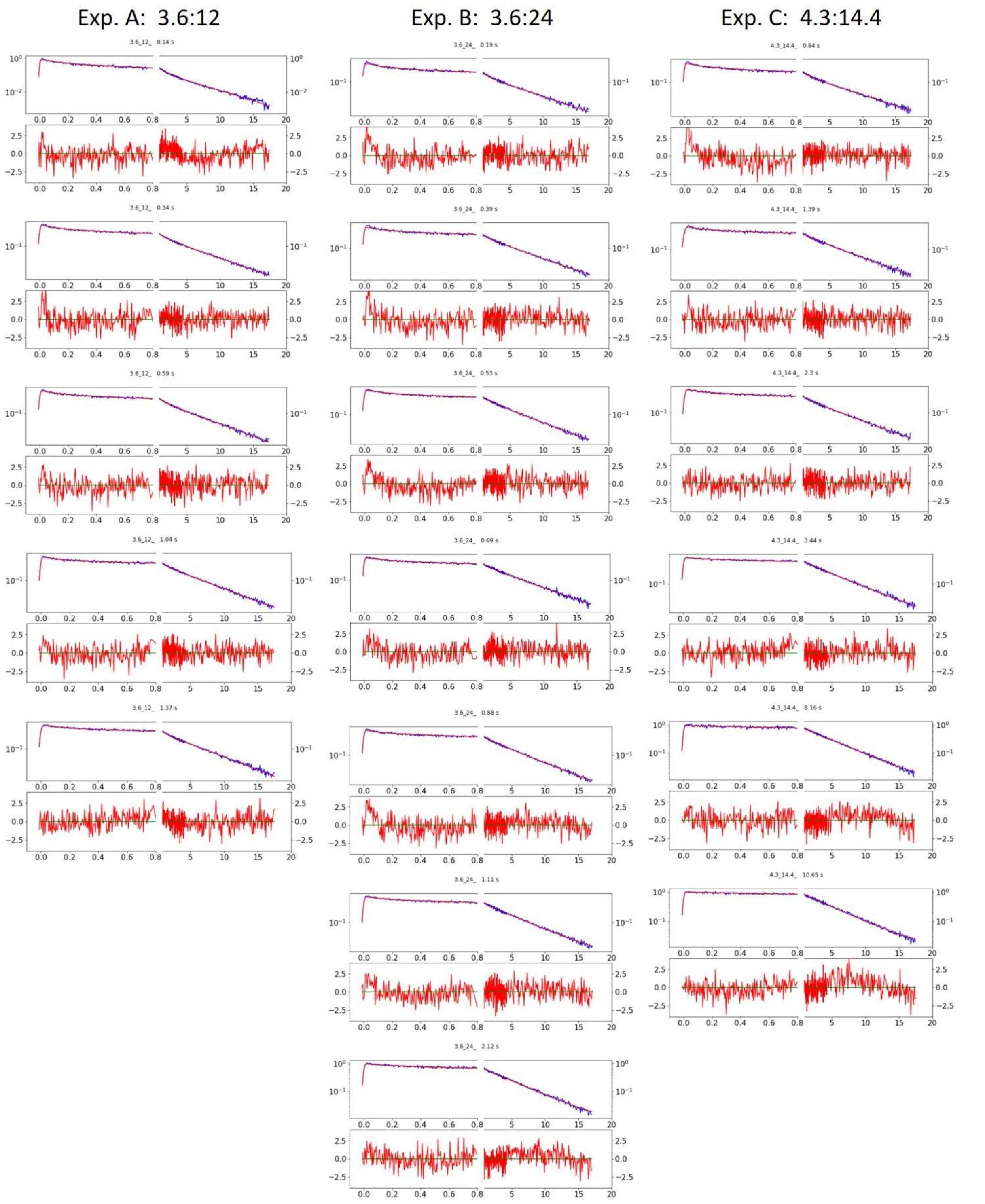

Figure S5: Preliminary analysis by a global fit with $F_1(t,T)$ (Eq. 5) of all decay kinetics recorded in three microfluidic experiments, “Exp. A”, “Exp. B”, “Exp. C”, displayed in three columns. In each column, the droplet propagation times T increase from top to bottom. At each propagation time, the recorded fluorescence decay kinetics (blue curve, horizontal axis t in ns) is fitted (overlapping red curve) with function $F_1(t,T)$ (Eq. 5), and the residuals are displayed below in red. Notice that in the first decays, the residuals indicate that a very short component - faster than 0.1 ns - is systematically not correctly fitted. We attribute it to a signature of IC, which is not considered by the fitting function $F_1(t,T)$. The reduced χ^2 of this global simultaneous fit of all displayed traces is 1.14.

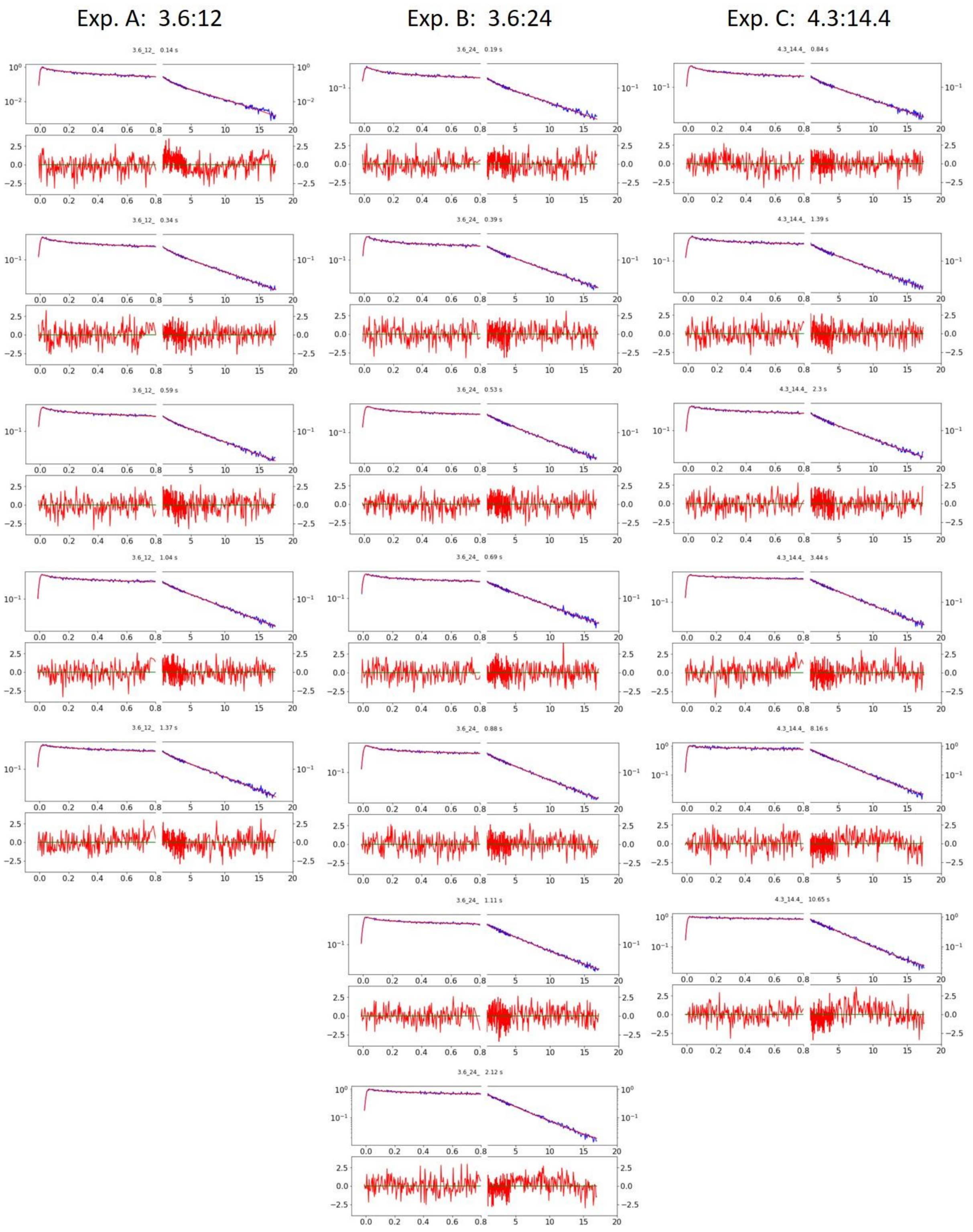

Figure S6: Global fit with $F_2(t,T)$ (Eq. 6). Same as Figure S5 except that the fitting function is now $F_2(t,T)$ (Eq. 6) in order to fit the fast component not captured in the preliminary fit displayed in Figure S5. The reduced χ^2 of the global simultaneous fit of all kinetics traces is now reduced to 1.05.

References

(1) Maillot, S.; Carvalho, A.; Vola, J.-P.; Boudier, C.; Mély, Y.; Haacke, S.; Léonard, J. Out-of-Equilibrium Biomolecular Interactions Monitored by Picosecond Fluorescence in Microfluidic Droplets. *Lab Chip* **2014**, *14* (10), 1767–1774. https://doi.org/10.1039/C3LC51283E.

(2) Bourbigot, S.; Ramalanjaona, N.; Boudier, C.; Salgado, G. F. J.; Roques, B. P.; Mély, Y.; Bouaziz, S.; Morellet, N. How the HIV-1 Nucleocapsid Protein Binds and Destabilises the (−)Primer Binding Site During Reverse Transcription. *Journal of Molecular Biology* **2008**, *383* (5), 1112–1128. https://doi.org/10.1016/j.jmb.2008.08.046.

(3) Bernacchi, S.; Mély, Y. Exciton Interaction in Molecular Beacons: A Sensitive Sensor for Short Range Modifications of the Nucleic Acid Structure. *Nucleic Acids Research* **2001**, *29* (13), e62–e62. https://doi.org/10.1093/nar/29.13.e62.

(4) Égelé, C.; Schaub, E.; Piémont, É.; de Rocquigny, H.; Mély, Y. Investigation by Fluorescence Correlation Spectroscopy of the Chaperoning Interactions of HIV-1 Nucleocapsid Protein with the Viral DNA Initiation Sequences. *Comptes Rendus Biologies* **2005**, *328* (12), 1041–1051. https://doi.org/10.1016/j.crvi.2005.06.005.

(5) Torimura, M.; Kurata, S.; Yamada, K.; Yokomaku, T.; Kamagata, Y.; Kanagawa, T.; Kurane, R. Fluorescence-Quenching Phenomenon by Photoinduced Electron Transfer between a Fluorescent Dye and a Nucleotide Base. *Analytical Sciences* **2001**, *17* (1), 155–160. https://doi.org/10.2116/analsci.17.155.
